# Reimagining early years services to address childhood inequities: learning from the Born in Bradford evaluation of a Better Start Bradford

**DOI:** 10.1136/bmjpo-2025-003995

**Published:** 2026-03-02

**Authors:** Josie Dickerson, Sara Ahern, Kate E Mooney, Sarah L Blower, Sunil Bhopal, Maria Bryant, Claudine Bowyer-Crane, Gill Thornton, Kerry Bennett, Sharon Goldfeld, John Wright, Kate E Pickett, Rosemary RC McEachan

**Affiliations:** 1 Bradford Institute for Health Research, Bradford, UK; 2 Department of Health Sciences, University of York, York, UK; 3 The Hull York Medical School, University of York, York, UK; 4 National Institute of Economic and Social Research, London, UK; 5 The University of Sheffield, Sheffield, UK; 6 Bradford Trident, Bradford, UK; 7 Centre for Community Child Health, Murdoch Childrens Research Institute, Parkville, Victoria, Australia; 8 Department of Paediatics, University of Melbourne, Parkville, Victoria, Australia

**Keywords:** Child Health, Epidemiology, Health services research, Health Policy

## Abstract

‘A Better Start’ was a 10-year (2015–2025), £215 million initiative funded by the National Lottery Community Fund, supporting five areas in England to address inequalities in the early years of life across socio-emotional development, language and communication, and nutrition outcomes. It aimed to provide a place-based, test-and-learn model, putting parents at the heart of design and delivery. As a result, each of the five sites developed and implemented distinct local programmes.

The Better Start Bradford programme delivered multiple preventative interventions across the outcome domains. Bradford was the only site to embed a research partner, Born in Bradford, from the very beginning. This enabled the establishment of a fully resourced research hub—the Better Start Bradford Innovation Hub, which included the world’s first interventional birth cohort, Born in Bradford’s Better Start, designed to efficiently evaluate multiple interventions simultaneously. This evaluation has provided in-depth learning about the inequalities faced in contemporary urban populations and evidence of the implementation and impact of multiple early years interventions.

In this review, we reflect on our ‘decade of discovery’: what worked well, what we have learnt about evaluating and delivering early years prevention at scale, and what we would do differently if we had the opportunity again. Examples of what worked well include the place-based model, the test-and-learn approach, a robust evaluation infrastructure and community empowerment. Our learning has evidenced important changes for future programmes and for commissioners, chief among them: complex inequalities cannot be resolved through the delivery of individual or scattered interventions. This collective learning points to a clear call for change to create a jointly commissioned, appropriately funded and continuously evaluated early years system, underpinned by a long-term commitment to *prevent* inequity in the early-years before it becomes entrenched.

**Key messages**

The Bradford experience offers key learning for other place-based community initiatives and highlights what must change to achieve sustained impact, including:

- Community empowerment and co-production enable families and stakeholders to influence decision making and co-create successful interventions, implementation plans and meaningful evaluations.
- An iterative test-and-learn approach allows for timely quality improvements and decommissioning of ineffective services.
- Using an equity lens ensures that appropriate interventions are implemented to address the local drivers of unequal outcomes.
- To effectively address inequities, a whole-system approach is needed with jointly commissioned, appropriately funded and continuously evaluated early years interventions.
- To reap the long-term economic and social benefits of early prevention, there must be a long-term policy commitment to protecting early years funding.

## A Better Start programme and Better Start Bradford

 In 2015, the National Lottery Community Fund implemented the ‘A Better Start’ (ABS) programme—a 10-year (2015–2025), £215 million initiative across five areas of England. The aim of the programme was ‘to improve the life chances of babies and very young children by changing the way services are commissioned and delivered, involving parents as equal partners’ and to do so by addressing four key outcomes: children’s nutrition, socio-emotional development, language and communication, and change in the early years ‘system’ (the way that local services work together).^1^

In England, proportionate universal services are provided to all families from birth to school age, including free access to midwifery and health visitor appointments, 30 hours of free Early Childhood Education and Care (ECEC) from age 3, and free education from age 4/5 onwards. For families who need additional support, more intensive support can be offered, for example, additional midwifery and health visitor appointments and access to Family Hubs. At the start of ABS (2015), Family Hubs were located in the heart of disadvantaged areas, offering an ideal place to reach families in need and provide additional support. ABS was designed to complement these proportionate universal services, offering add-on interventions to tackle the inequalities in health and development of children living in areas of high disadvantage.

Bradford was one of the areas which received this investment. Bradford is a large district in the North of England which has an ethnically diverse population covering rural, suburban and inner-city areas with areas of affluence and severe deprivation. 60% of children (4000 per year) in Bradford are born into families who live in areas within the highest decile of the Index of Multiple Deprivation in England. Local data show that half of these children were not ready to learn at school entry (as measured by the Early Years Foundation Stage Profile),^2^ meaning that at any one point in time, approximately 8000 families in the early years need additional support. The Better Start Bradford programme covered three of these highly deprived, inner-city areas of Bradford. In these areas, ~1400 babies are born each year and 88% of births are to parents from an ethnic minority background.^3^

Born in Bradford (BiB), an internationally recognised applied health research programme, worked as a key partner on the Better Start Bradford proposal to ensure there was a clear focus on evidence-based intervention. Bradford was the only ABS site to embed evaluation into its design from the beginning. This enabled a fully resourced, in-depth evaluation of the programme, including the world’s first interventional birth cohort—Born in Bradford’s Better Start (BiBBS), alongside a research hub (the Better Start Bradford Innovation Hub, hereafter the ‘Innovation Hub’) to support service design and complete implementation and qualitative evaluations. BiBBS built on the success of the BiB family cohort, recruiting pregnant women and their babies within maternity services using skilled community researchers, collecting in-depth information on the social determinants of health, biological samples and consent for linkage to routinely collected health, education and early years intervention data.^3 4^

This review will share reflections on the learning that the Innovation Hub has amassed throughout our decade of discovery. We will share the key strengths of the programme, what we have learnt about evaluating early years prevention at scale and, with the benefit of hindsight and advances in research approaches, what we would do differently if we had the same opportunity in the future.

## Strengths of the programme

### Service providers and the community as equal partners

Co-production has been a golden thread throughout Better Start Bradford and the Innovation Hub, ensuring that families and stakeholders have their voices heard and are able to influence, challenge and co-create relevant research and intervention delivery. BiB is a ‘people powered’ research programme, whose priorities are set by, and solutions to improve health and well-being co-produced by, families and stakeholders.^5^ A Community Research Advisory Group, consisting of local parents/carers and community members, was set up to support the design and interpretation of the research. The work of Better Start Bradford was similarly led by a multi-sectoral Partnership Board made up of senior leaders from across the early years system and an equal number of parents, who jointly drive the decision-making.

### Focus on reducing inequalities in the critical early years

There is unequivocal evidence that the earliest years of life, from birth to age three, are critical to a child’s developmental and health outcomes. What happens in these early years will have a lifelong impact on a child’s educational attainment, life opportunities and long-term physical and mental health.^6 7^ There is also clear evidence of inequities in the early years, with children living in areas of disadvantage and those from some ethnic minority backgrounds more likely to have early developmental delay and worse health.^6 8^ These inequities are entrenched, with more than a decade of data showing no improvements and the recent COVID-19 pandemic and cost-of-living crises further exacerbating inequities.^8 9^

Developmental and health inequities are driven by social determinants which intersect to create complex challenges for disadvantaged families at multiple levels—the family, the environment and the system.^10^ To change the developmental and health trajectories of children living in disadvantage, these drivers must be addressed. The implementation of multiple early preventative interventions to support families during pregnancy, birth, up to age 3, alongside programmes to improve the local environment and to influence the early years system was, therefore, a sensible approach by ABS.

### Place-based, test and learn approach

The intention of ABS was to deliver evidence-based preventative early interventions; however, in 2015, there was limited evidence about *how* to do this effectively^11^ and for those interventions that were backed by evidence, there was limited evidence of impact in real-world settings. Consequently, ABS proposed a pragmatic place-based, test and learn approach—delivering evidence-based interventions where possible (defined as tested and proven effective using robust study designs) as well as ‘science based’ interventions (defined as developed using the best available evidence but not tested or proven effective).^3^ In Bradford, only two evidence-based programmes were selected for delivery: The Family Nurse Partnership, whose evidence at the time came from outside the UK context^12^; and Incredible Years, whose evidence base was from older age groups.^13^ All other interventions were science-based but had been developed and/or delivered in the local context. The majority were universal interventions, with a small number of targeted interventions for families with specific needs (table 1). The structure of the programme was flexible, with families able to select to take part in universal interventions based on their preferences and be referred into targeted interventions.

**Table 1:** An overview of the Better Start Bradford interventions

Intervention	Timing	Description	Type	Take-up in BiBBS	Main outcome	Evidence level 2015	Evidence level 2025	System impact
Midwifery Continuity of Care	Pregnancy	Care by the same midwife/small team, longer appointment times and flexible visits	Universal	1020	Birth outcomes; socio-emotional development	4; NL2† (step 1)	4; 3 (2026)	RCT underway, ongoing delivery
ESOL+	Pregnancy	English language course for women with little or no English during pregnancy		41	Language and communication	NL2 (step 1)	NL2 (step 3)	No longer being delivered
Family Links Welcome to the World	Perinatal	Universal perinatal parenting programme	Universal	75	Socio-emotional development	NL2 (step 4)	NL2−	Decision to decommission
Family Nurse Partnership^1^	Perinatal	Intensive home visiting for vulnerable women aged <25	Targeted	32	Socio-emotional development	4	NL2−	Decision to decommission
Baby Steps	Perinatal	Perinatal parenting programme for vulnerable parents	Universal	492	Socio-emotional development	NL2 (step 4)	3 (2026)	Rolled out district-wide
Doulas	Perinatal	Late pregnancy, birth and post-natal 1 to 1 support for vulnerable women	Targeted	127	Socio-emotional development	NL2 (step 1)	NL2 (step 3)	Ongoing delivery
HAPPY	Perinatal	Healthy eating and parenting course for overweight mums with a BMI over 25	Targeted	170	Nutrition/health	3	NL2−	Decision to decommission
Family Action Perinatal Mental Health Support	Perinatal	Perinatal support for women at risk of mild/moderate mental health issues	Universal	93	Socio-emotional development	NL2 (step 2)	2 (2026)	Rolled out district-wide
Breast feeding support service	Birth	Universal practical and emotional support to breastfeeding mums and their families	Universal	1030	Nutrition/health	NL2 (step 1)	3 (2026)	Rolled out district-wide
MECSH	Perinatal	Enhanced home visiting programme delivered by health visitors to clients with additional needs	Targeted	Unk*	Socio-emotional; language and nutrition	3	3	Rolled out district-wide
Home-Start	Birth to 4	Peer support for vulnerable women	Targeted	79	Socio-emotional development	NL2 (step 1)	NL2 (step 3)	Ongoing delivery
Little Minds Matter	Birth to 2	Training and professional consultations on infant mental health; clinical support for those at risk	Targeted	Unk*	Socio-emotional development	NL2 (step 1)	NL2 (step 4)	Rolled out district-wide
Better Start Imagine	Birth to 3	Book gifting & book sharing sessions	Universal	2798	Language and communication	NL2 (step 1)	NL2 (step 4)	No longer being delivered
Better Place	Birth to 3	Structural improvements in parks and green spaces; interventions to encourage play	Universal	n/a	Physical activity/health	NL2 (step 1)	NL2 (Step 4)	Ongoing
Cooking for a Better Start	Age 1–3	Universal cook and eat sessions	Universal	107	Nutrition/health	NL2 (step 1)	NL2 (step 3)	No longer being delivered
HENRY	Age 1–3	Universal group programme to improve healthy eating and physical activity in young children	Universal	234	Nutrition/health	2	3 (2028)	Local QED and national RCT underway
Incredible Years- Toddler	Age 2–3	Universal parenting programme	Universal	187	Socio-emotional development	2	3 (2028)	Toddler model not being delivered
Preschoolers in the Playground	Age 2–3	Preschoolers’ physical activity in the playground	Universal	<5	Physical activity/health	2	NL2−	Decision to decommission
Forest Schools for Toddlers	Age 2–3	Outdoor play in the natural environment for young children attending nurseries, and parents	Universal	207	Socio-emotional development	NL2 (step 1)	3 (2027)	No longer being delivered
I CAN Early Talk	Age 2–3	Strengthening parents’ and practitioners’ knowledge in improving language development	Universal	n/a training in nurseries	Language and communication	NL2 (step 1)	NL2 (step 3)	No longer being delivered
Talking Together	Age 2	Universal screening; in-home programme for parents with children at risk of delay	Universal; targeted	2737; 622	Language and communication	NL2 (step 1)	3	No longer being delivered

* Unk—unknown number of participants at present time, as unable to access and link to individual data on project uptake.

† NL2 - Not yet Level 2 on the Early Intervention Foundation evidence ladder

BMI, body mass index; QED, quasi-experimental design; RCT, randomised controlled trial.

### Embedded rigorous evaluation programme

The embedding of the Innovation Hub into the programme design was a further key strength. This enabled a fully resourced, in-depth evaluation of the programme, funded at 20% of the total programme budget. It ensured that evaluation could be embedded as a part of the service design process to ensure high-quality implementation and effectiveness evaluations.

**Figure 1:**
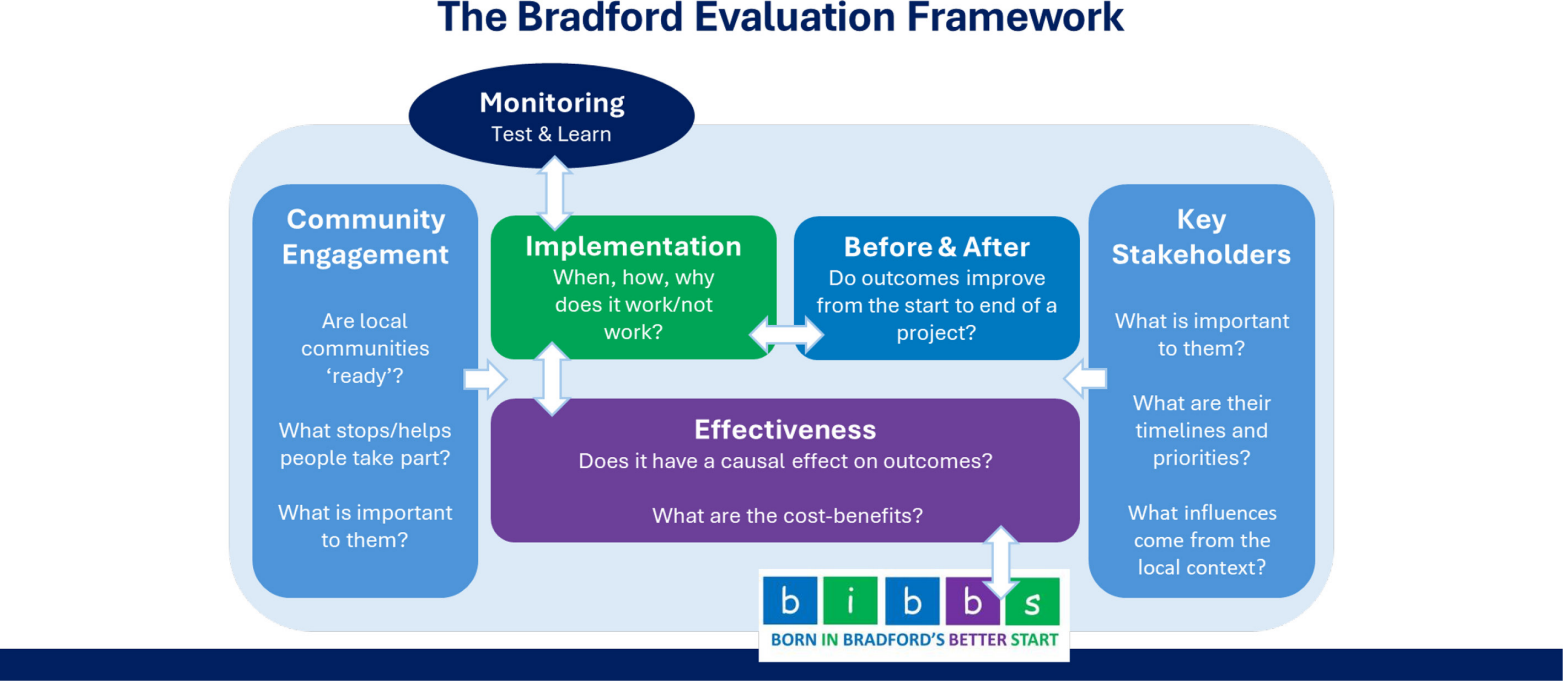
The Bradford evaluation framework.

Designing evaluations of multiple interventions that were all at differing levels of ‘readiness’ for evaluation, while also meeting the needs of local partners, the priorities of the community and the demands of high-quality academic research, was challenging. Over a 3-year period, we developed and refined a framework for evaluation within usual practice (figure 1), which recognised the community, stakeholders and local context as key influences on evaluation. The framework takes a staged approach to evaluation using the Early Intervention Foundation’s ‘evaluation ladder’^14^ to ensure that the planned evaluation ‘nudged up’ the intervention from its’ current level of evidence, and that timely evidence could be provided to commissioners and the community. The challenges of evaluation in practice and our solution using a pragmatic evaluation framework and associated toolkits have been described elsewhere.^15^ The success of the framework was due to how it: worked closely with the services to develop a theory of change and logic model; provided timely evidence on implementation success^16^ (ie, reach, engagement and fidelity of delivery) and evidence of promise (using ‘before and after’ evaluations and qualitative studies); and, for those interventions which demonstrated implementation success, delivered effectiveness evaluations using pragmatic randomised controlled trials and quasi-experimental methods.

This pragmatic approach allowed evidence-based interventions to be evaluated in areas of high need within the UK context, and science-based interventions to be evaluated to enhance their evidence of implementation and impact. In both instances, the provision of timely and dynamic learning enabled adaptations to be made where necessary, and for de-commissioning decisions to take place where interventions were shown to not be effective. This process was a key strength of the programme, allowing a cycle of learning and decision-making based on local evidence.

## What have we learnt about evaluating early years prevention at scale?

### Understanding the levels of need and the strengths of the community

BiBBS successfully recruited a representative sample of >5600 pregnant women and their babies into the cohort, which is a testament to the community involvement in the study design, the support of midwifery and interpreting services, and recruitment by skilled researchers who spoke community languages. Of these pregnancies, 62% were of Pakistani heritage, 10% were White British and 28% of mothers were from multiple other ethnic groups.^17^ While not representative of the UK, this cohort reflects the eligible population in Bradford and other disadvantaged inner-city populations, making it valuable locally, nationally and internationally. BiBBS was designed to efficiently evaluate multiple early years interventions, but it has also provided rich insight into the vulnerabilities and protective factors within this community, which has helped to refine intervention delivery and service provision over time. Key vulnerabilities include: 56% of mothers were born outside of the UK; 44% had English as a second language of whom 26% reported little or no English language ability^17^; 46% of mothers reported symptoms of depression and 30% symptoms of anxiety during pregnancy^4^; in children aged 2, 24% were at risk of language delay^18^ and 22% had developmental delay.^19^

Protective factors include the findings that, while families in the cohort live in areas of high deprivation, 75% reported being financially secure.^17^ Living in a larger household has been found to be protective against depressive symptoms in mothers during the pandemic^20^ and of a reduced risk of language delay in children.^18^

### Community readiness and engagement

At the start of the Better Start Bradford journey, the Innovation Hub undertook research using the Community Readiness Model to understand how ready and willing communities were to engage in interventions to address nutrition^21^ and socio-emotional development.^22^ The findings suggested low levels of readiness, which prompted a series of workshops to identify the barriers and enablers to engaging in the interventions using a socio-ecological model.^23^ The Innovation Hub, Better Start Bradford and community members then co-produced a community engagement framework based on three core principles of ‘Voice, Choice, and Power’.^24^ The framework emphasised the value of informal consultation, ongoing assessment of the community’s readiness to engage, and a flexible approach to ensure that interventions met the community’s needs. The inclusion of the community in governance roles was found to promote transparency, foster trusting relationships and build community capacity. This co-produced approach, anchored in a strong two-way feedback loop between the community and services, offers a transferable model for effective community engagement in diverse UK and global settings.^24^

### Evidence of intervention uptake

Of all the children in BiBBS, 84% had a parent/carer who attended one or more interventions,^4^ which is a great success in terms of the reach of the programme. Table 1 shows that most of this reach came through a small number of universal interventions, including a midwifery continuity of carer model, a book gifting scheme offered to all new babies, a breastfeeding peer support offer and a language screening assessment offered to all children aged 2. Other universal and targeted interventions had much smaller capacity and so were only able to reach a smaller proportion of families.^25^

**Figure 2:**
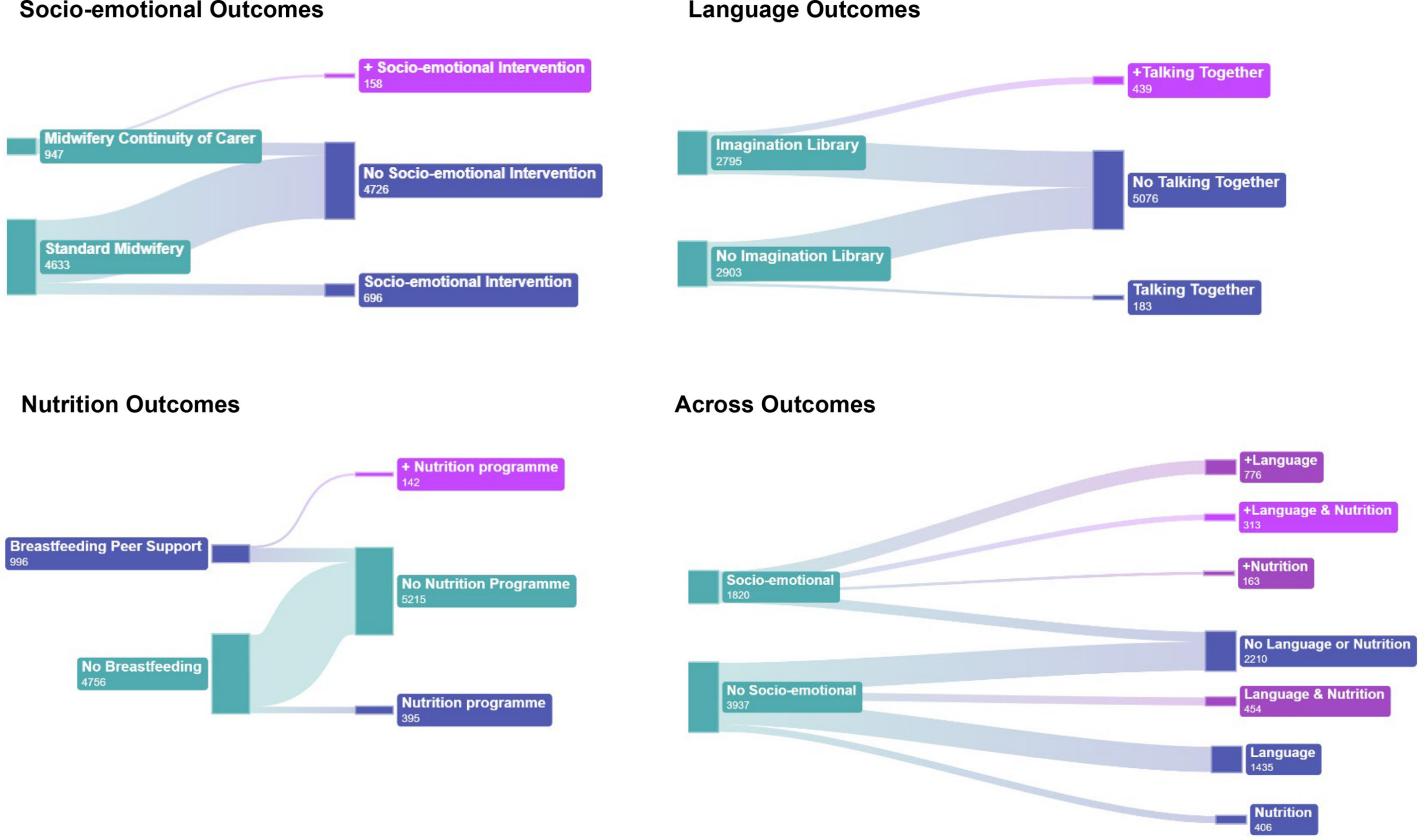
Engagement in combinations of interventions by outcome domain.

Of the parents/carers who attended an intervention, 57% attended two or more interventions. We have explored whether there are any patterns in the combinations of interventions that have been taken up by families. These combinations included participation in two or more interventions that aimed to improve one of the outcome domains, or participation in a combination of interventions across these outcomes (ie, *any* socio-emotional and *any* language and *any* nutrition intervention). These combinations and the number of families participating in each combination are shown in figure 2. In all instances, overlap of participation by BiBBS participants was small, ranging from 142 (two or more nutrition interventions) to 776 (two or more socio-emotional and language interventions).

The rich data in BiBBS have also allowed us to explore the intersectional characteristics that predict intervention uptake. These findings showed that mothers from an Asian/Asian British background, born outside of the UK, with good English language skills, but low levels of social support, were the most likely to take part in interventions. Those least likely to take part were mothers from Central/Eastern European ethnic backgrounds who were not born in the UK and had high levels of social support.^26^

### Evidence of implementation successes and failures

We have produced over 60 implementation evaluation reports providing evidence of the reach, recruitment rates, accessibility, acceptability and implementation characteristics for each intervention.^25^ These reports are publicly available to ensure other areas can benefit from this learning. The implementation evaluations have resulted in some interventions being decommissioned and alternatives being identified and delivered in their place, and others being scaled up to wider delivery. Examples of these include:

1. Perinatal parenting programmes. Initially, three perinatal parenting programmes were offered to pregnant women and their partners. Early implementation evaluations demonstrated the poor reach of two of these interventions (‘Welcome to the World’^27^ and HAPPY^28^), meaning that they could not be delivered with fidelity, while also demonstrating the success of one programme in terms of reach, engagement and completion (the Babysteps parenting programme^29^). Consequently, the two unsuccessful services were decommissioned and Babysteps received more funding to extend capacity. Since then, Babysteps has been adopted into mainstream commissioning across the city.
2. Enhanced support for vulnerable families. The Family Nurse Partnership programme is an intensive home-visiting intervention designed to support vulnerable, primarily young/single parents. The make-up of the Better Start Bradford areas, where most parents are older (mid-20s) and married, meant that there was not sufficient demand for this intervention to justify its high costs. During the ABS timeline, a trial demonstrated limited evidence of effect in the UK context.^30^ Together, this local and national evidence enabled a decommissioning decision to be made for this service, and an alternate, evidence-based programme more appropriate for this population—the Maternal Early Childhood Sustained Home-visiting programme^31^—to be implemented and evaluated. Following amendments recommended by the early implementation evaluation, the MESCH programme has also now been adopted into mainstream commissioning across the city.

### Effectiveness evaluations

The Innovation Hub has successfully implemented a platform for multiple effectiveness evaluations using BiBBS, also allowing a range of evaluation methods to be tried out on interventions delivered in usual practice (eg, trials within cohorts; pragmatic trials and quasi-experimental studies). Effectiveness evaluations only commenced once there was evidence of implementation success, ensuring efficiency by only evaluating interventions that could be successfully delivered in usual practice. However, this has meant that the evaluations have taken longer to commence than initially planned. Examples of these evaluations include:

1. A feasibility trial of an intervention to support families at risk of language delay (Talking Together), which demonstrated evidence of promise for improved parental warmth and vocabulary in the children.^32^
2. Three effectiveness evaluations are currently underway, looking at the impact of: the Babysteps perinatal parenting programme on perinatal mental health and the infant–child relationship^29^; the midwifery continuity of carer model on perinatal mental health and birth outcomes^33^; and a breastfeeding peer support programme on the duration of breastfeeding.
3. Three longer-term evaluations of the impact of: a toddler healthy eating programme (HENRY) on child body mass index aged 5; the Incredible Years Toddler parenting programme^34^ and a toddler Forest School intervention on socio-emotional development aged 5.

## What would we do differently if we had the same opportunity in the future?

Applied health research programmes are constantly evolving as learning and methods develop. The ABS programme enabled BiB to shift from a solution-focused observational cohort to an interventional cohort actively evaluating multiple interventions. From 2015 to 2025, there has been a further shift within BiB, and many other research programmes, to focus on a whole system’s approach, providing solutions to the upstream causes of inequities and a coordinated offer of interventions.^35^,^37^

This aligns with the growing recognition that interventions delivered in isolation cannot act as a ‘silver bullet’ to tackle the complex causes of developmental and health inequities. There is growing evidence that taking a complementarity approach, wherein families receive a combination or ‘stack’ of two or more early years interventions (such as those commissioned by Better Start), may show cumulative benefits on child outcomes, thereby reducing inequities throughout the life course.^38^,^40^

After a decade of learning through the Innovation Hub, alongside the shift to a whole system approach, we are now in a strong position to propose a pragmatic solution to tackle inequity in child development and health using an evidence-informed whole system approach in the early years. This includes the following requirements.

### Delivery of ‘stacks’ of preventative interventions

In Better Start Bradford, while multiple interventions were offered to families, their delivery was not coordinated—families were left to ‘pick and choose’ services to take part in.^3^ The finding described in this paper (see figure 2) that only small numbers of families participated in a combination of interventions suggests that, just as a silver bullet approach cannot work to improve entrenched inequities, a scattered approach also will not effectively engage families in multiple interventions to address the complex causes of inequities. If we want families to receive a coordinated ‘stack’ of interventions to effectively support all their needs and improve outcomes, then we must design the early years system to work in this way.^36^ This system also needs to include effective engagement strategies to ensure that all families can access and engage. It should also include embedded, appropriately funded evaluations—using our pragmatic evaluation framework to enhance the evidence base and encourage ongoing implementation and effectiveness evaluations in practice.

Although we do not yet have conclusive evidence of *which* precise combinations of services and interventions will enhance equitable outcomes, we propose that these stacks should include interventions that have evidence of: (a) engaging the populations that they need to reach; (b) being delivered with fidelity in practice *and* (c) being evidence-based or having evidence of promise. The stacks should: be delivered within existing services, including the Healthy Child Programme, ECEC and Family Hubs; offer additional universal interventions to families living in disadvantaged areas; cover financial well-being, socio-emotional development, language/communication and nutrition support.

In Bradford, this pragmatic approach is currently being co-produced to identify a solution that is acceptable to families, deliverable in practice and based on the best available evidence. This will include the consideration of how existing services can work together and share resources to successfully deliver this change. Advances in methodological approaches mean that we are also now preparing to evaluate the impact of the combinations of interventions in figure 2 using BiBBS data.

### Addressing the wider determinants of developmental and health inequalities

#### Child and family poverty

The ABS programme intentionally funded areas with high levels of economic disadvantage; however, the programme did not fund interventions to directly support families’ financial circumstances. Evidence shows the strong associations between child poverty and poor developmental and health outcomes across the lifespan.^6 8 39^ There is also evidence that financial hardship can restrict families’ abilities to support their children and engage in additional interventions.^40^ There are numerous potential evidence-based solutions to address poverty at a policy, environmental and family level. Examples include the Scottish child payment policy for families below a certain income threshold,^41^ and the healthier, wealthier families intervention providing non-stigmatised welfare and benefits support to families who are not receiving what they are entitled to.^42^

#### Environmental factors

A healthy urban environment is key to ensuring children can grow up to be healthy and happy; however, urban areas of disadvantage tend to include a high proportion of poor housing, overcrowding, high levels of air pollution and poor-quality green spaces. All these issues need to be addressed to support family health, and there are several evidence-based system-level interventions that can be implemented to achieve this.^43^ Better Start Bradford made significant infrastructure changes to local green spaces, offering safe and inviting places for children to play,^25^ and more widely in Bradford, a clean air zone was introduced in 2022, which has reduced air pollution and there is a suggested decrease in respiratory-related health service use.^43^ Such interventions demonstrate that investment in environmental-level interventions to reduce health inequalities is both possible and impactful.

### Longer-term policy commitment to early prevention

In the UK, policy and funding for early prevention tend to be tied to political cycles, and funding is susceptible to cuts in times of economic crises. Since the ABS programme began, there have been significant cuts in national spending on early years services through austerity. From 2011/2012 to 2021/2022, Local Authority cuts in early intervention services, including Family Hubs, dropped by 46%^44^; and the Healthy Child Programme had a reduction of £850 million in investment.^45^ The ABS programme’s mission to provide additional complementary services to address inequalities has become increasingly challenging to achieve in a system struggling with a loss of workforce, service capacity and infrastructure. In contrast, the evidence of impacts of such preventative interventions is seen much further into the future as children reach health and developmental milestones. An example of this is the Family Hubs, where the evidence of significant impacts on health and educational inequalities emerged after investment in the service was stripped back.^44^

In 2026, re-investment in preventative interventions in the early years is a top priority for the UK Government^46 47^ offering the potential for our learning to be mobilised into practice across England. However, it is also imperative for this investment to be protected in order to reap the longer-term economic benefits across all public sector services. To do so, there needs to be longer-term funding commitments more akin to the commitment to infrastructure investments, such as energy/transport. Such investment also needs to acknowledge the scale of the problem—to give all children living in disadvantage an equitable start in life will require significant investment, especially in areas of high need. For example, at any one time, there are ~8000 children aged 0–4 living in severe deprivation in Bradford alone who would benefit from our proposed early years support system.

#### Embedded evaluations

The evaluation of Better Start Bradford provides a decade of learning that shows early years interventions can be designed and implemented to reach diverse and disadvantaged families. We have shown that embedded research can help inform the effectiveness of these interventions and decommission interventions that do not show promise. A recognition of the value of long-term evaluations within future programmes, embedded from the very beginning and properly costed in, is crucial to add much-needed evidence of what works, and for whom, in the early years of life.

## Conclusion

Better Start Bradford—and its research partnership with BiB—offer a number of key lessons for other community-based initiatives on how to enhance equitable outcomes in early childhood development. These include: the value of community empowerment and co-production to ensure that families and stakeholders influence decision making and co-create successful interventions, implementation plans and meaningful evaluations^5 24^; the benefits of an iterative test-and-learn approach to enable timely quality improvements and de-commissioning decisions for ineffective services^3 15^ and how taking an equity lens to local data ensures that appropriate interventions are implemented that address the local drivers of unequal outcomes and use local assets to protect against inequalities.^10 25^

The Bradford experience shows what is possible but also highlights what must change to achieve sustained impact within a generation. It is clear that no single intervention can tackle inequities in child development and health. Similarly, a scattered approach is not enough to ensure that families receive combinations of interventions to address the drivers of inequities. To improve outcomes, a coherent, whole system approach to the early years is needed, including: a pathway of stacked interventions; supported by upstream system interventions and policy changes; complemented by community engagement; and pragmatic, embedded evaluation and learning.^35 36^ Such an approach may feel radical, but based on the learning from the evaluation of Better Start Bradford, we now know it is an achievable solution to give all children the best possible start in life.
