# Supplementary material for: Reimagining early years services to address childhood inequities: learning from the Born in Bradford evaluation of a Better Start Bradford
Source: BMJ Paediatr Open. 2026 Mar 2;10(1):e003995. doi: 10.1136/bmjpo-2025-003995 (PMC12983683; doi:10.1136/bmjpo-2025-003995)
Supplement: Supplementary file 1 [file bmjpo-2025-003995.reviewer_comments.pdf]

PEER REVIEW HISTORY

BMJ Paediatrics Open publishes all reviews undertaken for accepted manuscripts. Reviewers are asked to complete a checklist review form and are provided with free text boxes to elaborate on their assessment. These free text comments are reproduced below.

ARTICLE DETAILS

**TITLE (PROVISIONAL)**	Reimagining Early Years Services to Address Childhood Inequities : Learning from the Born in Bradford Evaluation of A Better Start Bradford
**AUTHORS**	Dickerson, Josie<br>Ahern, Sara<br>Mooney, Kate<br>Blower, Sarah Louise<br>Bhopal, Sunil<br>Bryant, Maria<br>Bowyer-Crane, Claudine<br>Thornton, Gill<br>Bennet, Kerry<br>Goldfeld, Sharon<br>Wright, Prof John<br>Pickett, Kate E<br>McEachan, Rosemary RC

VERSION 1 – REVIEW

**REVIEWER NAME**	Bird, Christopher
**REVIEWER AFFILIATION**	Birmingham Women's and Children's NHS Foundation Trust, Emergency Department
**REVIEWER CONFLICT OF INTEREST**	No Conflict of Interest
**AI DISCLOSURE**	No, I have not used AI
**DATE REVIEW RETURNED**	15-Jan-2026

**GENERAL COMMENTS**	Dear Dr Dickerson et al,<br><br>Thank you for this very helpful and timely review at a time when health and social care are trying to navigate a move from hospital to community, make good on NHS aims to integrate care and address health equity but with very limited resources. There are several important observations for commissioners, health and social care professionals delivering services for CYP and the communities that should be working alongside them, in particular:<br><br>1) the importance of factoring in sufficient resource to evaluate interventions and to ahve these embedded from the start<br>2) the learning around what works and what doesn't and the importance of an iterative approach, decommissioning or modifying services that aren't making an impact based on these evaluations (test-and learn)<br>3) the importance of co-production - that without this, there is unlikely to be good take-up of services<br>4) I found the idea of "stacked interventions", citing research from Australia, particularly useful

	I have some very minor suggestions:<br>1) page 10, lines 36-38 - references to the studies described here<br>2) on page 11, lines 10-15, I would suggest referencing MRC guidance on evaluating complex interventions, which i think relevant here (https://www.bmj.com/content/374/bmj.n2061)<br>3) given the importance the authors give to stacked interventions, it would be worth a line or two and references to the literature around the possible value - but also the challenges - of integrating care for CYP, e.g. Wolfe I, Satherley RM, Scotney E, Newham J, Lingam R. Integrated Care Models and Child Health: A Meta-analysis. Pediatrics. 2020 Jan;145(1):e20183747. doi: 10.1542/peds.2018-3747 or Baxter S, Johnson M, Chambers D, Sutton A, Goyder E, Booth A. The effects of integrated care: a systematic review of UK and international evidence. BMC Health Serv Res. 2018 May 10;18(1):350. doi: 10.1186/s12913-018-3161-3.<br><br>Also, Table 1 is a useful reference and there are cited examples of where the teams decided to stop services that weren't working but I wonder if this might be better presented as a couple of case study boxes, one for an intervention that didn't work and one for where there was evidence of impact.

**REVIEWER NAME**	Lenton, Simon
**REVIEWER AFFILIATION**	
**REVIEWER CONFLICT OF INTEREST**	No Conflict of Interest
**AI DISCLOSURE**	No, I have not used AI
**DATE REVIEW RETURNED**	22-Jan-2026

**GENERAL COMMENTS**	Review. Reimagining Early Years Services to Address Childhood Inequities: Learning from the Born in Bradford Evaluation of A Better Start Bradford<br><br>Summary.<br>This article is a reflection on learning from the implementation and evaluation of the “A Better Start” from the academic recipients of the £215 million initiative to address inequalities in the preschool period in Bradford, UK over a ten-year period.<br>Novelty.<br>It is good to receive this paper which reflects and learns from the experience of implementing and evaluating multiple intervention simultaneously in real life scenarios. This work is not only important for the future academic evaluation of similar programmes but there are important messages here for those commissioning (planning), delivering and evaluating “everyday” services within future integrated care systems.<br>Major comments.<br>BMJ Paediatrics Open seeks to publish papers that will positively influence the future development of services that improve overall equity and outcomes.<br>This paper could be published in its current form; however, I believe the key messages and conclusion sections could be improved with a summary of the learning more applicable to existing community-based preschool services, in areas not supported by a well-resourced research team. Examples might include:<br>• Taking a whole system approach to the future development of services to create alignment, synergy and continuity between the various parts contributing to improving equity of outcomes.

	Reference Royal Academy of Engineering. Engineering Better Care (addresses the silver bullet versus scattergun approaches)<br>• The importance of participation and coproduction between the users of the services and the various stakeholders involved with the delivery of services (two-way communication/feedback) Reference Council of Europe Child Friendly Healthcare.<br>• The importance of developing measures that reflect both the delivery (structure and process) and the results (outputs, outcomes and impact (in this case equity)) that are of value to multiple stakeholders (meaningful, measurable, motivating and able to monitor sustainability and fidelity. Reference Public Health Observatory<br>• Embedding a culture of continuous learning within integrated power system (reference Don Berwick A Promise to Learn). Important learning between ICSs NHS England Improving Networks.<br>• The importance of being able to decommission less effective in order to reinvest in more effective ways of working based on local evaluation.<br>• The importance of both protection (from hazards, harmful to health) and promotion of assets (factors that contribute to well-being and resilience) when planning interventions related to the life course pathway and the equity of outcomes.<br><br>Minor Comments.<br>• Proportionate universalism may require explanation for non-UK readers.<br>• Reference needed Good Level of Development (GLD) and School readiness.<br>• Substitute “social determinants of health” with “health determinants” (the paper recognises the importance of economic, the physical environment and access to services which are wider than just social relationships between people.<br>• MECSH programme needs expansion to Maternal Early Childhood Sustained Home-visiting (MECSH) programme<br>• “Alignment and synergy between services” might be a better phrase than a “stack” of interventions.<br>• Reference to the NHS Ten-Year Plan where it stated the intention is to “create the healthiest generation of children ever”.<br>• Figure 1. The Bradford evaluation framework diagram is not a descriptive “logic model” based on pathway delivery described in the review.<br>• Figure 2. Requires explanation!<br>• Table 1. I presume this will be a web link is too detailed for paper publication.

VERSION 1 – AUTHOR RESPONSE

Reviewer One

Thank you for taking the time to review this paper, and for your positive comments. We have resolved the minor comments made as follows:

1) page 10, lines 36-38 - references to the studies described here

Relevant references have been added, and the wording has also been altered to make it clear that this sentence is referring to the findings shared in Figure 2 of this paper (unpublished data).

2) on page 11, lines 10-15, I would suggest referencing MRC guidance on evaluating complex interventions, which i think relevant here (https://www.bmj.com/content/374/bmj.n2061)

Thank you, this reference has been added in.

3) given the importance the authors give to stacked interventions, it would be worth a line or two and references to the literature around the possible value - but also the challenges - of integrating care for CYP.

We haven't added in these references as integrated care pathways (changing the way medical services work together and are delivered) are quite different to what we mean by the stacking of community interventions (changing the way individual interventions are offered). Instead we have re-worded this sentence to make what we mean by "stacks" clearer.

4) Changing Table 1 to case-studies.

This is a great idea but loses much of the detail available in Table 1. There are, however, short descriptions of two such case studies in the section on "Evidence of implementation successes and failures".

Reviewer 2:

Thank you for taking the time to review this paper and for your comments on adding value to the key messages and conclusions which are much appreciated. We have addressed your points as follows:

Major comments

BMJ Paediatrics Open seeks to publish papers that will positively influence the future development of services that improve overall equity and outcomes.
This paper could be published in its current form; however, I believe the key messages and conclusion sections could be improved with a summary of the learning more applicable to existing community-based preschool services, in areas not supported by a well-resourced research team …

Thank you for these observations highlighting the learning for community-based services in the key messages and conclusions. We have now added these into both relevant sections. We have chosen to use the references that are already in the text for each of these points as we feel these are the most relevant to this review, and the number of references is already at the maximum limit. However, if the reviewer / editor deems these important we are very happy to add them in.

Minor Comments.
• Proportionate universalism may require explanation for non-UK readers.

The wording in this paragraph has been amended to explain this term more clearly.

• Reference needed Good Level of Development (GLD) and School readiness.

This has now been added in.

• Substitute “social determinants of health” with “health determinants” (the paper recognises the importance of economic, the physical environment and access to services which are wider than just social relationships between people.

We prefer the term social determinants and the reference we use to such a model (ref 10) also uses this term to describe the determinants at the level of the family, the environment and the system.

• MECSH programme needs expansion to Maternal Early Childhood Sustained Home-visiting (MECSH) programme

This has now been properly named.

• “Alignment and synergy between services” might be a better phrase than a “stack” of interventions.

“Stacks” are a phrase used in the early years’ literature (e.g., refs 37-40) so we have chosen to keep this, but have re-phrased this sentence to make this definition clearer.

• Reference to the NHS Ten-Year Plan where it stated the intention is to “create the healthiest generation of children ever”.

This reference has been added alongside a reference to the UK Government Best Start Plan.

• Figure 1. The Bradford evaluation framework diagram is not a descriptive “logic model” based on pathway delivery described in the review.

Figure 1 is described in the text as an evaluation framework. The success of the framework includes a range of toolkits including logic model development and we believe this is clear in the text.

• Figure 2. Requires explanation!

We have added a description to the Figure

• Table 1. I presume this will be a web link is too detailed for paper publication.

We’ll leave this to editorial decision
